# Magnetic resonance metabolic profiling of estrogen receptor-positive breast cancer: correlation with currently used molecular markers

**DOI:** 10.18632/oncotarget.18822

**Published:** 2017-06-28

**Authors:** Ji Soo Choi, Dahye Yoon, Ja Seung Koo, Siwon Kim, Vivian Youngjean Park, Eun-Kyung Kim, Suhkmann Kim, Min Jung Kim

**Affiliations:** ^1^ Department of Radiology, Breast Cancer Center, Samsung Medical Center, Seoul, Korea; ^2^ Department of Chemistry, Center for Proteome Biophysics and Chemistry Institute for Functional Materials, Pusan National University, Busan, Korea; ^3^ Department of Pathology, Severance Hospital, Yonsei University College of Medicine, Seoul, Korea; ^4^ Department of Forensic Chemistry, National Forensic Service Busan Institute, Yangsan-si, Korea; ^5^ Department of Radiology, Research Institute of Radiological Science, Yonsei University College of Medicine, Seoul, Korea

**Keywords:** breast cancer, ER-positive, luminal, HR-MAS MRS (high-resolution magic angle spinning magnetic resonance spectroscopy), biomarker

## Abstract

Estrogen receptor (ER)-positive breast cancers overall have a good prognosis, however, some patients suffer relapses and do not respond to endocrine therapy. The purpose of this study was to determine whether there are any correlations between high-resolution magic angle spinning (HR-MAS) magnetic resonance spectroscopy (MRS) metabolic profiles of core needle biopsy (CNB) specimens and the molecular markers currently used in patients with ER-positive breast cancers. The metabolic profiling of CNB samples from 62 ER-positive cancers was performed by HR-MAS MRS. Metabolic profiles were compared according to human epidermal growth factor receptor 2 (HER2) and Ki-67 status, and luminal type, using the Mann-Whitney test. Multivariate analysis was performed with orthogonal projections to latent structure-discriminant analysis (OPLS-DA). In univariate analysis, the HER2-positive group was shown to have higher levels of glycine and glutamate, compared to the HER2-negative group (*P*<0.01, and *P* <0.01, respectively). The high Ki-67 group showed higher levels of glutamate than the low Ki-67 group without statistical significance. Luminal B cancers showed higher levels of glycine (*P*=0.01) than luminal A cancers. In multivariate analysis, the OPLS-DA models built with HR-MAS MR metabolic profiles showed visible discrimination between the subgroups according to HER2 and Ki-67 status, and luminal type. This study showed that the metabolic profiles of CNB samples assessed by HR-MAS MRS can be used to detect potential prognostic biomarkers as well as to understand the difference in metabolic mechanism among subtypes of ER-positive breast cancer.

## INTRODUCTION

Since it became known that a large subset of breast cancers depends on estrogen receptor (ER) signaling, leading to the clinical application of endocrine therapies (e.g., tamoxifen, aromatase inhibitors), breast cancers have been classified into ER-positive and ER-negative cancers [1–5]. With appropriate endocrine therapy, patients with ER-positive cancers have significantly better outcomes than those with ER-negative cancers [1–4]. Subsequent gene expression profiling studies of ER-positive breast cancers demonstrated that this group is still heterogeneous in ER expression levels and proliferation-related genes [6, 7], which is associated with clinical outcomes and treatment response. Currently, ER-positive cancers are subclassified into luminal A and luminal B types based on human epidermal growth factor receptor 2 (HER2) overexpression and Ki-67 labeling index using a cut-off of 14% [8]. Luminal B cancers, high proliferative ER-positive cancers, have a poor prognosis compared to luminal A [9, 10]. This “intrinsic gene” molecular subclassification of ER-positive cancers has been widely used by clinicians to predict prognosis and to select treatment options for patients with these cancers [8, 11].

In addition to the genetic alteration of cancer, metabolic alteration of cancer is also important to understand the cancer biology and to find potential biomarkers that can be targeted therapeutically [12, 13]. Metabolic alterations are the consequence of genetic alterations in metabolic pathways and are directly linked to cell phenotype [14]. Accordingly, the metabolite levels of the cells provide functional changes associated with cell metabolism [12, 15, 16]. Metabolomics is defined as the study of all of the metabolites of a cell, tissue or organism for a comprehensive understanding of metabolism [12, 14, 16, 17]. Recent metabolomic studies have shown that *ex vivo* high-resolution magic angle spinning magnetic resonance spectroscopy (HR-MAS MRS) can be used for the identification and quantification of numerous metabolites in a tissue sample [12, 16, 18–23]. Compared to other metabolomic approaches, HR-MAS MRS requires less sample treatment and does not damage tissue integrity after the experiment, thus allowing reuse of the tissue sample for other diagnostic examinations. In HR-MAS MRS studies using human breast tissue, surgical specimens or core needle biopsy (CNB) specimens were used [18–23]. Metabolic profiles of CNB samples are applicable to preoperative decision-making for the best treatment approach for breast cancer patients, whereas metabolic profiles of surgical samples are not. Previous HR-MAS MRS studies using CNB samples have reported that metabolic profiles of CNB samples may be helpful in the diagnosis and characterization of breast cancer, and monitoring of responses to neoadjuvant chemotherapy in locally advanced breast cancer [20, 21, 24].

Profiling intrinsic gene expression and metabolite content in the same breast cancer tissue could reveal differences and similarities in the metabolic composition between groups of samples at different gene expression levels and provide increased insight into functional changes that are potential targets for pharmacological or nutritional intervention [25]. Although recent studies have compared metabolic profiles between triple-negative breast cancers and ER-positive breast cancers [18, 26], no HR-MAS MRS studies, to our knowledge, have examined the association between metabolic and molecular markers only in ER-positive breast cancers. The comparison between metabolic profiles and expression levels of HER2 and Ki-67, which are used in molecular classification of ER-positive breast cancer tissue, may help identify potential biomarkers associated with clinically aggressive subgroups. Therefore, the purpose of this study was to determine whether there are any correlations between HR-MAS MRS metabolic profiles of CNB specimens and molecular markers currently used in patients with ER-positive breast cancers.

## RESULTS

The median age of the study population was 51.5 years (range, 32-84 years; interquartile range [IQR], 45-60 years). The median tumor size was 1.6 cm (range, 0.5-4.2 cm; IQR, 1.2-2.2cm). The most common histologic type of ER-positive cancers was invasive ductal carcinoma (n=57) and other cancers included three mucinous carcinomas and two invasive lobular carcinomas.

All study patients received proper management after surgery and underwent postoperative surveillance with US or mammography after initial treatment. The follow-up period ranged from 35-72 months (median, 42 months; interquartile range, 39.5-68 months). None of the patients had a breast cancer recurrence during the follow-up period.

**Figure 1:**
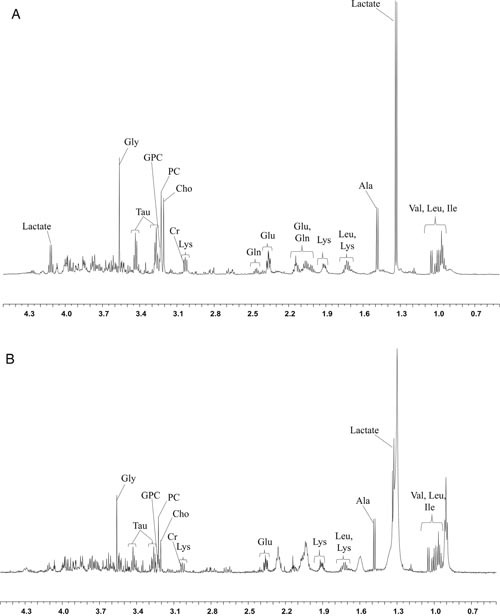
The HR-MAS MR spectra (11.7T) obtained using core needle biopsy specimens show the peaks of each metabolite **(A)** A 48-year-old woman with luminal B type ER-positive invasive ductal carcinoma (tumor size 17 mm, presence of lymph node metastasis, HER2-positive, High Ki-67). **(B)** A 57-year-old woman with luminal A type ER-positive invasive ducal carcinoma (tumor size 15 mm, no lymph node metastasis, HER2-negative, Low Ki-67). Gly, glycine (3.55 ppm, singlet); Tau, taurine (3.24 and 3.41 ppm, triplet); GPC, glycerophosphocholine (3.22 ppm, singlet); PC, phosphocholine (3.21 ppm, singlet); Cho, free choline (3.20 ppm, singlet); Cr, creatine (3.03 ppm, singlet); Lys, lysine (3.01 triplet,/1.72 and 1.89 ppm, multiplet); Gln, glutamine (2.15 and 2.44 ppm, multiplet); Glu, glutamate (2.08 and 2.34 ppm, multiplet); Leu, leucine (1.69 ppm multiplet/0.91 and 0.94 ppm, doublet); Ala, alanine (1.47 ppm, doublet); Val, valine (0.98 and 1.04 ppm, doublet); Ile, isoleucine (0.99 ppm, triplet/1.02 ppm doublet).

As shown in Table 1, relative quantification showed that several metabolite levels of CNB tissue samples were significantly different between the groups. The HER2-positive group was characterized by higher levels of Gly and Glu, compared to the HER2-negative group (*P*<0.01 and P<0.01 respectively). The HER2-positive group showed higher level of Cho, without reaching statistical significance (*P*=0.04). The high Ki-67 group showed higher level of Glu than the low Ki-67 group, with borderline significance (*P*=0.02). Luminal B cancers showed significantly higher levels of Gly (*P* =0.01) than luminal A cancers (Figure 1). Luminal B cancers showed higher levels of PC and Tau (*P*=0.03, and *P* =0.05, respectively) and lower levels of Ile (*P* =0.04) than luminal A cancers (Figure 1), although the differences did not reach statistical significance.

**Table 1:** Comparison between the relative metabolite quantification levels of ER-positive breast cancers according to the tumor groups

	HER2			Ki-67			Molecular subtype*		
	Negative (n=52)	Positive (n=10)		Low (n=39)	High (n=23)		Luminal A (n=36)	Luminal B (n=26)	
Metabolite	Median (IQR)	Median (IQR)	*P*	Median (IQR)	Median (IQR)	*P*	Median (IQR)	Median (IQR)	*P*
Cho	1.16 (0.88-1.71)	1.74 (1.34-2.05)	0.04	1.33 (0.85-1.74)	1.32 (1.00-1.79)	0.60	1.30 (0.83-1.71)	1.48 (1.00-1.92)	0.07
PC	1.15 (0.69-1.49)	1.51 (1.35-1.75)	0.08	1.09 (0.67-1.59)	1.41 (1.18-1.58)	0.27	1.08 (0.58-1.43)	1.41 (1.18-1.62)	0.03
GPC	0.46 (0.35-0.70)	0.43 (0.30-0.57)	0.14	0.46 (0.29-0.71)	0.47 (0.36-0.56)	0.48	0.46 (0.30-0.70)	0.45 (0.36-0.57)	0.42
Gly	**6.97 (5.28-8.71)**	**9.60 (8.51-9.93)**	**<0.01**	7.05 (5.14-8.86)	8.54 (6.47-9.68)	0.15	**6.89 (5.02-8.63)**	**8.56 (6.51-9.69)**	**0.01**
Ser	5.57 (4.35-6.79)	5.90 (5.40-6.42)	0.30	5.60 (4.04-6.86)	5.75 (4.66-6.46)	0.32	5.52 (4.11-6.79)	5.81 (4.65-6.49)	0.67
Tau	4.91 (2.92-6.07)	5.87 (5.36-6.85)	0.10	4.96 (2.87-6.08)	5.71 (3.99-6.52)	0.77	4.91 (2.77-5.99)	5.75 (4.11-6.95)	0.05
Leu	3.51 (3.03-4.06)	3.36 (2.98-4.44)	0.86	3.51 (3.04-7.03)	3.41 (2.89-4.33)	0.79	3.58 (3.13-4.06)	3.38 (2.87-4.30)	0.39
Ile	1.65 (1.41-2.10)	1.46 (1.29-1.76)	0.10	1.66 (1.42-2.30)	1.56 (1.36-1.79)	0.20	1.68 (1.43-2.34)	1.57 (1.36-1.76)	0.04
Gln	1.90 (1.47-2.24)	2.02 (1.40-2.39)	0.35	1.98 (1.43-2.25)	1.93 (1.42-2.36)	0.98	2.04 (1.60-2.26)	1.89 (1.35-2.33)	0.33
Glu	**6.18 (5.03-6.78)**	**7.46 (6.30-9.15)**	**<0.01**	6.07 (4.82-6.73)	6.64 (5.93-8.25)	0.02	6.13 (4.97-6.77)	6.55 (5.88-8.24)	0.19
Cr	1.17 (0.82-1.77)	1.13 (0.74-1.49)	0.34	1.16 (0.75-1.83)	1.17 (0.87-1.72)	0.32	1.12 (0.72-1.80)	1.17 (0.91-1.74)	0.78
m-Ins	1.79 (1.43-2.61)	2.44 (2.09-2.89)	0.05	2.10 (1.43-2.67)	2.03 (1.55-2.55)	0.87	2.08 (1.43-2.80)	2.05 (1.54-2.57)	0.51
Ala	5.52 (4.44-6.64)	6.78 (5.74-7.82)	0.17	5.50 (4.40-6.72)	6.23 (5.02-6.96)	0.80	5.46 (4.45-6.37)	6.26 (4.91-7.21)	0.11

Cho: choline; PC: phosphocholine; GPC: glycerophosphocholine; Gly: glycine; Ser: serine; Tau: taurine; Gly: glycine; Leu: leucine; Ile: isoleucine; Gln: glutamine; Glu: glutamate; Cr: creatine; m-Ins: myoinositol; Ala: alanine.

*St Gallen surrogate molecular subtype.

IQR: interquartile range.

**Bold** indicates statistical significance (*P*<0.0167).

**Figure 2:**
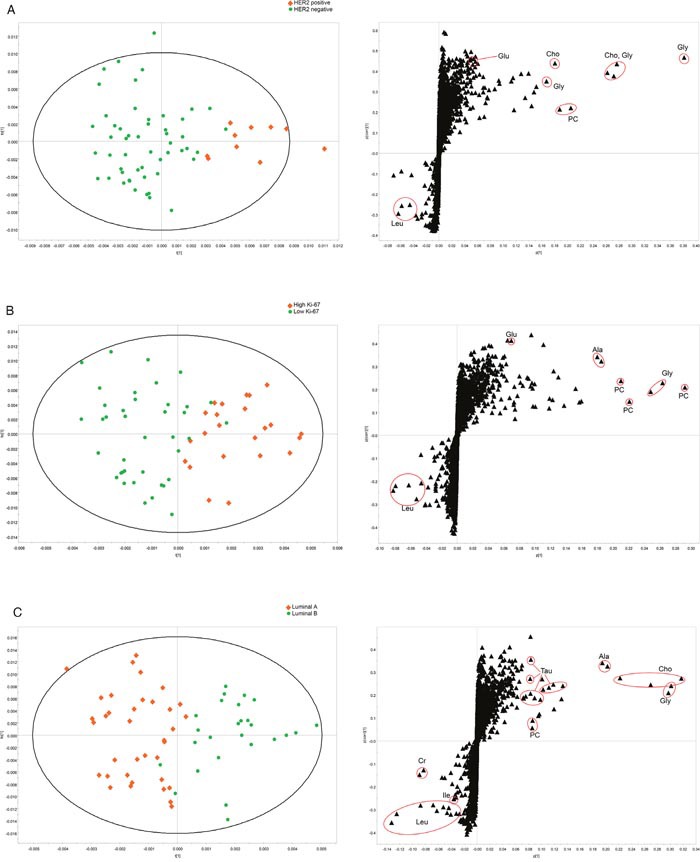
OPLS-DA score and loading S-plots of the HR-MAS MRS spectra for HER2, Ki-67 status, and luminal type **(A)** HER2-positive vs. HER2-negative, **(B)** high Ki-67 vs. low Ki-67, and **(C)** luminal A vs. luminal B.

For multivariate analysis, OPLS-DA separation models were produced with the HR-MAS MRS data according to HER2 and Ki-67 status, and luminal type. The OPLS-DA models showed visible discrimination between the groups, although some samples crossed over the reference line (Figure 2). Corresponding OPLS-DA loading S plots showed that Gly, PC, Cho, and Tau were contributing metabolites for prediction of the groups of ER-positive cancers. In addition, Leu contributed to discrimination of HER2-negative, low Ki-67, and luminal A groups from corresponding groups, although the level of Leu showed no statistical significance in univariate analysis. OPLS-DA prediction models of this study showed high sensitivities with a range of 94.4 −100% for prediction of HER2, Ki-67, and luminal type (Table 2).

**Table 2:** OPLS-DA classification results of HER2, Ki-67 status, and molecular subtype

	Sensitivity	Specificity	Accuracy
HER2-negative vs. HER2-positive	100.0 %	71.2 %	75.8 %
Ki-67 low vs. Ki-67 high	95.8 %	82.1 %	88.7 %
Luminal A vs. Luminal B	94.4 %	92.3 %	93.5 %

Orthogonal projections to the latent structure discriminant analysis (OPLS-DA) classification models were built to separate the tumor groups according to the metabolic profiles of the CNB samples. The statistical relevance was verified using “Y-scrambling” validation, and the model was validated by prediction of unknown samples using the leave-one-out method.

For calculating diagnostic values, positive results of the OPLS classification models were defined as HER2-positive, high Ki-67, and luminal B cancers, respectively.

## DISCUSSION

Patients with ER-positive breast cancers are generally thought to have a good prognosis, but some experience recurrence while others have cancers that are unresponsive to endocrine therapy [8, 25]. Metabolic profiling of ER-positive cancers may help to detect biomarkers that can be used to identify aggressive subgroups. Recent studies have performed metabolic profiling of ER-positive breast cancers using surgically obtained tissue [18, 25–27]. However, metabolic profiles of surgical samples cannot be obtained preoperatively for planning of therapeutic strategies. On the other hand, metabolic profiles of cancer samples obtained by US-guided CNB, standard procedure for preoperative diagnosis of breast cancers [28], are applicable to preoperative decision-making for the best treatment approach. In addition, several studies have reported that some protein and phosphoprotein levels were significantly higher in CNB compared with surgical samples suggesting a potential degradation of phosphorylation during surgical manipulation, or with cold ischemia of surgical samples [29, 30]. We have previously investigated HR-MAS MRS data of CNB breast cancer samples, and the results showed that several metabolites were found to correlate with IHC status of tumor [20]. In that study, ER-negative cancers showed significantly higher levels of Cho than ER-positive cancers. However, the study population of this past study included only 28 cases of ER-positive cancers, and we did not perform subgroup analysis according to molecular subtype. Considering that ER-positive cancers make up approximately 70% of breast cancers and that treatment outcomes differ according to molecular subtype (luminal A vs. B) [31], we attempted to conduct HR-MAS MRS metabolic profiling with a larger number CNB samples of ER-positive cancers in this study.

HER2 overexpression is associated with more aggressive disease and poorer prognosis compared to HER2 negativity [32]. Patients with HER2-positive cancers have been shown to be effectively treated by HER2-targeted therapy (e.g., trastuzumab) [33]. Accordingly, HER2 status evaluation is currently used to classify ER-positive cancers into luminal A or B type to guide therapeutic strategies. In this study aimed at ER-positive breast cancers, the HER2-positive group and luminal B group showed significantly higher levels of Gly compared to the HER2-negative group and luminal A group, respectively. This is in accordance with a recent study in which there was a positive association between Gly level in surgical specimens and HER2-positive cancers [18]. Gly is an amino acid involved in the synthesis of proteins, nucleotides and glutathione, and higher levels of Gly have previously been found to correlate with rapid cell proliferation and poor prognosis in breast cancer [22, 27, 34]. In addition, a recent study reported that glycine decarboxylase (GLDC), which is associated with Gly metabolism, was highly expressed in HER2-positive cancers [35]. Although the precise mechanism of increased GLDC in HER2-positive cancers is unknown, a 20-fold increase in GLDC expression was seen in MCF10A cells after oncogenic transformation by KRAS^G12D^, PIK3CA^E545K^, or MYC^T58A^ [36], suggesting that HER2-positive cancer may be driven by certain HER2 oncogenes. Therefore, based on results from previous studies combined with our results, we suggest that Gly may have potential as a prognostic biomarker that reflects tumor aggressiveness associated with HER2 overexpression in ER-positive cancers as well as HER2-positive subtype of breast cancer.

Along with HER2-positivity, a high Ki-67 proliferation index is associated with worse survival outcome in breast cancer patients [37]. Our high Ki-67 and HER2-positive aggressive subgroups of ER-positive cancer showed significantly higher levels of Glu compared to the corresponding groups, although Gln levels were not significantly different. Gln provides nitrogen for protein and nucleotide synthesis and Gln addiction has been described as a main feature of cancer cell metabolism [38]. Our findings may reflect an early step in increased glutaminolysis in which Gln is converted to Glu in the cytosol or mitochondria [38]. This finding is in accordance with a recent study in which Glu enrichment was found in 56% of ER-positive cancers and in 88% of ER-negative cancers, compared with normal breast tissue. Other recent studies have reported increased glutaminolysis to be more prominent in ER-negative or triple negative cancers compared to ER-positive cancers [18, 39]. Along with previous studies, our results indicate that targeting the metabolites or enzymes related to glutanimolysis metabolism may provide a new therapeutic strategy for the aggressive subgroups of ER-positive cancers or ER-negative cancers.

Choline-containing metabolites including Cho, PC, and GPC are associated with cell signaling, lipid metabolism and cell membrane synthesis and degradation [40]. In our study, Cho and PC levels were higher in the HER2-positive and luminal B subgroups of ER-positive breast cancer. However, the differences in these metabolite levels did not reach statistical significance, which may be due to the relatively lower levels of choline-containing metabolites of ER-positive cancers compared to ER-negative cancers [20, 41]. Nonetheless, the tendency of higher levels of PC and Cho in the HER2-positive and luminal B subgroups of our ER-positive cancer samples is in accordance with those from recent metabolomics studies using CNB specimens or surgical tissue which found significant correlation between choline-containing metabolites in breast cancer tissue and the aggressive subgroups of breast cancer [18, 20]. These results may be a consequence of the up-regulation of choline kinase activity, which is associated with tumor aggressiveness and drug sensitivity [34, 40, 42, 43]. Down-regulation of choline kinase has been shown to decrease cell proliferation and to increase the effect of chemotherapy in breast cancers [42, 43]. Therefore, we suggest that choline-containing metabolites may be biomarkers related to the aggressive subgroups of ER-positive cancers.

In our OPLS-DA analysis, higher levels of Leu were associated with the less aggressive groups (HER2-negative, low Ki-67, and luminal A) of ER-positive cancers, although the levels of Leu did not reach statistical significance in univariate analysis. In addition, the luminal A group showed higher levels of Ile compared to luminal B. Although little is known about the relationship between breast cancer and branched-chain amino acids (BCAA) including Leu, Ile, and valine, a recent study has reported that plasma levels of Leu and BCAA (sum of Ile, Leu, and valine levels) were significantly correlated with the level of free testosterone in premenopausal women [44]. Leu and testosterone levels in plasma have been associated with obesity and insulin resistance [45, 46], which are identified as risk factors for breast cancer [47]. Considering these findings, one possible hypothesis for higher levels of BCAA in the less aggressive subgroups of our ER-positive cancers is that a subgroup with obesity or insulin resistance may be associated with better prognosis than a subgroup without. So, higher levels of BCAA may be a marker of patient obesity or insulin resistance, not a prognostic marker. To our knowledge, this study was the first to suggest the Leu level in CNB samples as a biomarker for ER-positive cancers. Therefore, additional studies are needed to evaluate whether BCAA can be appropriate biomarkers associated with the prognosis of ER-positive cancers.

OPLS-DA multivariate models using HR-MAS MRS data of pretreatment CNB cancer samples provided visible discrimination between the luminal A and luminal B groups, which supports the usefulness of molecular subclassification of ER-positive cancers widely used in clinical practice. The diagnostic accuracy for predicting the luminal type was higher than those for predicting the groups classified by a single factor (HER2 and Ki-67, respectively). Our results suggest that OPLS-DA multivariate analysis using HR-MAS MRS metabolic profiles of CNB samples may provide comprehensive prognostic information reflecting molecular features of ER-positive cancers. Therefore, prognostic information obtained with HR-MAS MRS metabolic profiles may help define a clinical subgroup of ER-positive cancers for which aggressive therapy such as additional cytotoxic chemotherapy should be considered over conventional treatment. In addition, the metabolites that contribute to the prediction of luminal type (Gly, PC and Leu) in univariate and OPLS-DA analyses can be more powerful targets for metabolic drugs. Based on our results, HR-MAS MRS metabolic profiles of CNB samples are thought to potentially help clinicians develop more personalized treatment protocols for ER-positive cancers.

This study had several limitations. First, we could not evaluate associations between metabolic profiles of samples and long-term outcomes of study patients, because no recurrences were observed during the follow-up period. Recently, ESR1 gene mutations have been found in 15-20% of patients with endocrine-resistant metastatic ER-positive cancers, the majority of which are treated with endocrine therapy [48, 49]. Therefore, we think that further research evaluating the metabolic profiles of ER-positive cancers with ESR1 gene mutations will be needed to identify potential biomarkers associated with poor prognosis of patients with ER-positive cancers. Second, we did not evaluate the relationship between the metabolic profiles and tumor-stroma ratios of our CNB samples, although recent studies have reported that the tumor-stroma ratio of breast cancer is associated with the prognosis [50, 51]. Third, there has been concern that biospecimen type (*in vivo* CNB sample vs. *ex vivo* surgical sample) may affect biomarkers or metabolic profiling. However, we did not compare HR-MAS MRS data obtained with CNB specimens with the data obtained with surgical specimens. Therefore, we cannot exclude the possibility that HR-MAS MRS metabolic profiles of the CNB specimen may not fully represent the metabolic composition of the tumors with heterogeneous histologic features, although a recent study reported that most individual proteomic biomarkers studied did not differ according to biospecimen type [29]. Fourth, we used TSP as an internal reference for metabolite quantification. Although TSP has been commonly used as a reference substance in HR-MAS MRS experiments [18–22, 24, 25, 52, 53], it can potentially bind to some proteins [54]. Therefore, we tried to compensate for this binding effect by taking the protein-bound TSP signal into account when setting up the TSP signal as our reference value for metabolite quantification. In spite of this effort, we could not rule out the possibility of unwanted errors from pathological differences between the cancer samples [55]. Thus, we used metabolite levels calculated by the relative quantification method in our comparison analysis. Finally, we used surrogate IHC measurement instead of multi-gene molecular assays for subtype classification of ER-positive cancers. Although multi-gene molecular assays were recognized as providing accurate and reproducible prognostic information, surrogate IHC-based classification was more widely applicable at a lower cost and was more readily available [8].

In conclusion, metabolic profiles of CNB samples of ER-positive breast cancers showed significant correlation with HER2, Ki-67, and luminal type. Our results indicate that the metabolic profiles of CNB samples assessed by HR-MAS MRS can be used to detect potential prognostic biomarkers as well as to understand the difference in metabolic mechanism among subtypes of ER-positive breast cancer.

## MATERIALS AND METHODS

### Patient and sample preparation

The institutional review board of Severance Hospital, Yonsei University College of Medicine, approved this prospective study, and written informed consent was obtained from all patients. Between December 2011 and December 2013, we initially enrolled 97 patients with 100 breast lesions assessed by the Breast Imaging Reporting and Data System as final assessment categories 4c or 5 [56], and larger than 1 cm in diameter on mammography or ultrasound (US). Finally, 61 patients with 62 breast lesions fulfilled the following inclusion criteria: (a) having a breast lesion pathologically diagnosed as ER-positive cancer by CNB, (b) not pregnant at the time of diagnosis, and (c) no history of breast cancer or previous breast surgery including breast implants. All patients were treated with surgery and seven patients underwent neoadjuvant chemotherapy (NAC) prior to surgery.

For pathologic diagnosis of each patient, US-guided CNB was performed with a 14-gauge dual-action semiautomatic core biopsy needle (Stericut with coaxial guide; TSK laboratory, Tochigi, Japan) by one of four radiologists (with 7-15 years of experience in breast imaging) before treatment (surgery or NAC prior to surgery). The radiologists targeted the homogeneously solid area for biopsies of large and heterogeneous lesions. An average of six (range 5-8) tissue samples were acquired by US-guided CNB. Leaving out one core sample of each lesion, the rest of samples were taken for histopathologic diagnosis and immunohistochemical (IHC) analysis. The core sample was put in a cryogenic vial and immersed in liquid nitrogen immediately after biopsy for HR-MAS MRS. These samples were kept in a freezer (MVE Cryosystem, Chart BioMed, CA, USA) at −162°C for one to five months before the HR-MAS MRS experiment.

### Pathologic analysis

For primary breast cancers, final histopathologic results of CNB and surgical specimens were reviewed to determine final diagnosis, tumor size, and molecular subtype determined by ER, progesterone receptor [PR], HER2, and Ki-67 status. Axillary lymph node status was determined by surgical histopathologic results and preoperative fine-needle aspiration biopsy results.

ER and PR positivity were defined using a cutoff value of > 1% positively stained nuclei [57]. HER2 staining using the Hercep Test TM (DAKO, Glostrup, Denmark) was interpreted as 0, 1+, 2+, or 3+ according to the guidelines of the American Society Clinical Oncology/College of American Pathologists [58]. Tumors scored as 3+ were considered HER2-positive cases whereas tumors with 0 to 1+ were regarded as negative cases. Tumors scored as 2+ required further investigation using fluorescence *in situ* hybridization to assess HER2 gene amplification. Ki-67 staining was scored by counting the number of cells with positively stained nuclei and was expressed as a percentage of the total number of tumor cells. Ki-67 results were classified into low (<14%) and high (≥14%) [59].

### HR-MAS MRS

Frozen CNB samples were thawed in the nuclear magnetic resonance (NMR) laboratory, weighed, and placed in a HR-MAS nanoprobe® (Agilent, Walnut Creek, CA, USA). The samples (mean weight 10 mg) were cut to fit a 40-μl NMR nanotube, and they were placed in the cell with the remaining volume filled with D_2_O containing 2 mM trimethylsilyl propionic acid (TSP) for chemical shift referencing. HR-MAS MR spectra were acquired with an NMR spectrometer (Agilent, VNMRS 600) operating at a proton NMR frequency of 600 MHz (11.74 T). An inverse-detection type probe equipped with a single Z gradient coil was used and the temperature was set to 26°C after calibration with methanol. The spectral acquisition parameters were as follows: CPMG (Carr-Purcell-Meiboom-Gill) pulse sequence to impose a T2 filter {[recycle delay-90°-(τ-180°-τ) 80-acquisiton] (pw90=6.0 us, τ =469.0 us)}, spinning rate of 2 kHz, 19.231 K complex data points, 9615.4 Hz sweep width, 2.0-s acquisition time, 1.0-s relaxation delay, 1.5-s saturation time, 256 number of transients, and total acquisition time of 16 min 18 sec.

Following acquisition, the spectra were processed and analyzed using ACD software (Advanced Chemistry Development, Toronto, Ontario, Canada) followed by post-processing steps of Fourier transformation, phasing and baseline correction. TSP was calibrated to 0.00 ppm, and spectral region from 0.5 to 7.6 ppm was chosen as the final input data analysis. Signals of chemical contamination (e.g., ethanol), water, and lipids were excluded ahead of the analysis. The peak amplitude of each metabolite was measured by fitting a Voigt (e.g., Gauss+Lorentz) line-shape function. Relative metabolite quantification was carried out based on the comparison between the integrated TSP signal and the signal of interest in the sample spectrum.

### Follow-up

Curative breast surgery was performed on all study patients and seven patients underwent NAC prior to surgery. Adjuvant endocrine therapy and/or chemotherapy were performed according to the pathologic characteristics of the cancers [60]. All patients were examined with mammography and US every 6 months for the first 2 years after surgery and annual mammography and US thereafter. Locoregional recurrence limited to the ipsilateral breast or chest wall and/or regional lymph node recurrence (axillary, infraclavicular, or supraclavicular lymph nodes), contralateral breast cancer, and distant metastasis to other body organs were regarded as breast cancer recurrence) [61].

### Data and statistical analysis

Pathologic characteristics of the included tumors were collected from a review of patient medical records, and are listed in Table 3. Tumor size was determined as the maximum diameter of invasive tumor on final pathologic results (n=55). However, tumor size measured with US was used for patients treated with neoadjuvant chemotherapy (n=7). For statistical analysis, tumors were grouped by status of HER2, and Ki-67, and St Gallen surrogate molecular subtype (luminal A or luminal B) [20, 22]. The Kolmogorov–Smirnov test was used to check for normal distribution of relative quantification levels of metabolites of CNB samples. The relative quantification levels of the metabolites (alanine [Ala], creatine [Cr], free choline [Cho], phosphocholine [PC], glycerophosphocholine [GPC], glutamine [Gln], glutamate [Glu], leucine [Leu], isoleucine [Ile], myo-inositol [m-Ins], taurine [Tau], serine [Ser] and glycine [Gly]; Supplementary Table 1) were compared according to the tumor groups using the Mann-Whitney test. Statistical analysis was performed with SAS for Windows, version 9.0 (SAS Institute, Cary, NC, USA). A *P* value of less than 0.0167 (Bonferroni corrected *P*=0.05/3) was considered to indicate a significant difference between groups.

**Table 3:** Correlation of molecular and pathologic characteristics of 62 ER-positive breast cancers in this study

	Tumor size		Lymph node metastasis	
	<2cm (n=46)	≥ 2cm (n=16)	Negative (n=40)	Positive (n=22)
HER2-negative (n=52)	39	13	32	20
HER2-positive (n=10)	7	3	8	2
Ki-67 low (n=39)	32	7	21	18
Ki-67 high (n=23)	14	9	19	4
Luminal A (n=36)	29	7	20	16
Luminal B (n=26)	17	9	20	6

Data present number of cancers.

For multivariate analysis, Matlab (MathWorks, Natick, MA), SIMCA-P+ 12.0 (Umetrics, Sweden), and Excel (Microsoft, Seattle, WA) programs were used. Orthogonal projections to latent structure discriminant analysis (OPLS-DA) were conducted to separate the tumor groups with metabolic profiles of CNB samples by building the statistical models. The statistical relevance was verified using “Y-scrambling” validation, and the resulting R^2^ and Q^2^ values were calculated. The R^2^ value represents the “goodness of fit” which reflects how close the calculated data are to the original data. By repeated fitting, the R^2^ value can be artificially increased, leading to a decrease in the Q^2^ value, the predictability. Therefore, statistical soundness can be checked by randomly permutating the group designation of the original data and seeing if the calculation can correctly differentiate the groups with this deliberately generated model. The model was validated by prediction of unknown samples using a leave-one-out method [62]. An a priori cut-off value of 0.5 was applied to evaluate the prediction results [63]. Signals contributing to group discrimination were detected by an S-plot and the corresponding spectral data were extracted from Chenomx (Spectral database; Edmonton, Alberta, Canada) software and an in-house data repository. Any signals related to contamination (e.g. ethanol and methanol) were removed from the statistical analysis of spectral data.

## SUPPLEMENTARY TABLE



## Abbreviations

ER: estrogen receptor
HR-MAS MRS: high-resolution magic angle spinning magnetic resonance spectroscopy
CNB: core needle biopsy
PR: progesterone receptor
HER2: human epidermal growth factor receptor 2
OPLS-DA: orthogonal projections to latent structure discriminant analysis
US: ultrasound
NAC: neoadjuvant chemotherapy
IHC: immunohistochemical
NMR: nuclear magnetic resonance
TSP: trimethylsilyl propionic acid
Cr: creatine
Ala: alanine
Cho: free choline
PC: phosphocholine
GPC: glycerophosphocholine
Gln: glutamine
Glu: glutamate
Leu: leucine
Ile: isoleucine
m-Ins: myo-inositol
Tau: taurine
Ser: serine
Gly: glycine
GLDC: glycine decarboxylase
BCAA: branched-chain amino acids
