# Supplementary material for: Magnetic resonance metabolic profiling of estrogen receptor-positive breast cancer: correlation with currently used molecular markers
Source: Oncotarget. 2017 Jun 28;8(38):63405–16. doi: 10.18632/oncotarget.18822 (PMC5609932; doi:10.18632/oncotarget.18822)
Supplement: Supplementary file 1 [file oncotarget-08-63405-s001.pdf]

Magnetic resonance metabolic profiling of estrogen receptor-positive breast cancer: correlation with currently used molecular markers

SUPPLEMENTARY MATERIALS

REFERENCES

1. Mishra P, Ambs S. Metabolic signatures of human breast cancer. Mol Cell Oncol. 2015; 2.
2. Griffin JL, Shockcor JP. Metabolic profiles of cancer cells. Nat Rev Cancer. 2004; 4:551-561.
3. DeBerardinis RJ, Chandel NS. Fundamentals of cancer metabolism. Sci Adv. 2016; 2:e1600200.
4. Hicks DG, Kulkarni S. HER2+ breast cancer: review of biologic relevance and optimal use of diagnostic tools. Am J Clin Pathol. 2008; 129:263-273.
5. Wise DR, Thompson CB. Glutamine addiction: a new therapeutic target in cancer. Trends Biochem Sci. 2010; 35:427-433.
6. Babchia N, Calipel A, Mouriaux F, Faussat A-M, Mascarelli F. The PI3K/Akt and mTOR/P70S6K signaling pathways in human uveal melanoma cells: interaction with B-Raf/ERK. Invest Ophthalmol Vis Sci. 2010; 51:421-429.
7. Monirujjaman M, Ferdouse A. Metabolic and physiological roles of branched-chain amino acids. Adv Mol Biol. 2014; 2014.
8. Moran J, Hernandez-Pech X, Merchant-Larios H, Pasantes-Morales H. Release of taurine in apoptotic cerebellar granule neurons in culture. Pflugers Arch. 2000; 439:271-277.
9. Duffy MJ. Serum tumor markers in breast cancer: are they of clinical value? Clin Chem. 2006; 52:345-351.
10. Barcelos R, Stefanello S, Mauriz J, Gonzalez-Gallego J, Soares F. Creatine and the liver: metabolism and possible interactions. Mini Rev Med Chem. 2016; 16:12-18.

Supplementary Table 1: Metabolites analyzed in this study

Metabolite	Physiologic function
Choline (Cho), Phosphocholine (PC), Glycerophosphocholine (GPC)	Cell signaling, lipid metabolism, cell membrane synthesis and degradation [1–4]
Glutamine (Gln), Glutamate (Glu)	Synthesis of proteins and lipids, alternative cellular energy source to glucose [1, 5]
Glycine (Gly)	Synthesis of proteins, nucleotides and glutathione [1–3]
Serine (Ser)	Synthesis of proteins, nucleotides, precursor to several amino acids including glycine [1–3]
Leucine (Leu), Isoleucine (Ile)	Promotes protein synthesis and turnover, signaling pathways, and metabolism of glucose [6, 7]
Taurine (Tau)	Antioxidation, osmoregulation and volume regulation [2, 8, 9]
Creatine (Cr)	Recycling of adenosine triphosphate, energy currency of the cell [10]
Myo-inositol (m-Ins)	Osmoregulation and volume regulation [2]
Alanine (Ala)	Synthesis of proteins, link to glycolysis and gluconeogenesis [2]
